# First Episode of Psychosis in the Context of Varenicline Treatment for Smoking Cessation

**DOI:** 10.7759/cureus.36677

**Published:** 2023-03-25

**Authors:** Yuri Zoghbi, Raja Barazi, Menatalla H Abdelkader, Yassin H Eltorki, Suhaila Ghuloum

**Affiliations:** 1 Psychiatry, Hamad Medical Corporation, Doha, QAT; 2 Pharmacy, Hamad Medical Corporation, Doha, QAT

**Keywords:** medication induced psychosis, adverse drug reactions, nicotine agonist, smoking cessation, first episode psychosis, psychotic disorder, varenicline

## Abstract

Smoking is one of the leading causes of preventable death worldwide. Several pharmacological therapies have been established over the years to aid in smoking cessation, including Varenicline, a partial nicotine agonist. Neuropsychiatric adverse events have been reported in patients treated with Varenicline. Here, we present a case of first-episode psychosis in the context of Varenicline therapy.

The patient’s chart was retrospectively reviewed for relevant medical and psychiatric history, in addition to the use of current or past medications. Routine laboratory investigations and brain imaging were performed. The Naranjo Adverse Drug Reaction Probability Scale was also done independently by two physicians involved in the patient’s treatment.

He was admitted for psychotic symptoms elicited in the context of a probable adverse reaction to Varenicline. The current evidence linking Varenicline to psychosis remains controversial. Speculatively, there could be a link between Varenicline, which supposedly increases dopamine levels in the prefrontal cortex through the mesolimbic pathways, and psychotic symptoms. It is therefore beneficial to be cognizant, in a clinical setting, of the possibility of the emergence of these symptoms with Varenicline therapy.

## Introduction

Smoking is the leading cause of preventable death worldwide because of its association with many complications, including cancer, heart disease, pulmonary disease, and premature death [1]. When nicotine binds to the neuronal α4 β2 receptor, dopamine is released in the nucleus accumbens, the reward center of the brain, which is believed to be responsible for the reinforcing or addictive properties of nicotine [2]. In order to tackle nicotine dependence, both non-pharmacological and pharmacological therapies have been established over the years, the latter including mainly nicotine replacement therapy (NRT), varenicline, and bupropion [2,3].

Varenicline is a partial nicotine agonist that was approved in the UK in the last decade for the treatment of smoking cessation in adults. Cessation rates and long-term abstinence ratios have been higher with the use of varenicline compared to the use of bupropion, nicotine replacement therapy, or placebo [4]. Moreover, in a recent meta-analysis of 20 randomized control trials, the combination of varenicline with other available interventions was associated with a better effect on smoking cessation. [5] Varenicline prevents nicotine stimulation of the mesolimbic dopamine system, which is associated with nicotine addiction, by partially binding to the neuronal α4 β2 receptor. In addition, it stimulates dopamine activity, resulting in decreased craving and withdrawal symptoms, which might facilitate the success of a cessation attempt [2]. Since varenicline acts on the dopaminergic pathways in the central nervous system, it is feasible to appreciate an impact on mood and suicide risk.

During clinical trials, varenicline was usually well tolerated, with nausea being the most commonly reported adverse event. Neuropsychiatric adverse events were seldom reported in patients without and with pre-existing psychiatric disease; some patients experienced worsening of their psychiatric illnesses [6-8]. For instance, mood changes, including depression and mania, psychosis, hallucinations, paranoia, delusions, homicidal ideation, aggression, hostility, agitation, anxiety, panic, suicidal ideation, suicidal attempt, and completed suicide, were noted in Food and Drug Administration (FDA) post-marketing reports, but the frequency of these adverse effects was not defined [8]. Despite symptoms' persistence in some cases, complete resolution of symptoms after discontinuation of varenicline has been reported [9].

It remains advisable for healthcare providers to monitor patients for the emergence of neuropsychiatric adverse events and consider a dose reduction of varenicline or medication discontinuation based on the severity of the symptoms and the extent to which the patient is benefiting from treatment [9].

In this paper, we present a case of first-episode psychosis in the context of treatment with varenicline for smoking cessation aid in the state of Qatar.

## Case presentation

We present this case of a 27-year-old unmarried male with no known past psychiatric or family history who presented to the psychiatric emergency department via emergency medical services due to a report of reckless behavior with an acute onset of psychotic features. This gentleman was found roaming the streets among traffic and was noted to display aggressive outbursts towards anyone who attempted to intervene on site. The patient necessitated multiple chemicals and restraints upon the arrival of the ambulance team, including 100 mg of ketamine and three doses of 2 mg of midazolam IM, before he reached a state of feasible conversation. He exhibited poor reality testing, poor insight, and poor judgment at the time of evaluation.

Collateral history revealed that the altercation was initiated by the patient with security staff members at an entrance to a mosque within a private accommodation complex. The events escalated to an aggressive outbreak, with the patient removing his shirt in public and responding with irrelevant speech.

Upon arrival at the emergency department, the physical assessment was compromised due to the patient's uncooperation; however, the airway was maintained with no signs of respiratory distress. The patient was conscious, oriented, hemodynamically stable, and moving all limbs, with routine vital assessments maintained within the normal range.

Laboratory workup, including a complete blood count, complete metabolic panel, urine illicit drug screen, and thyroid function test, were assessed and found to be unremarkable. In addition, a brain CT scan was also conducted with no significant findings.

On initial psychiatric assessment in the emergency department, the patient was reported to have irrelevant speech, a labile mood, and persecutory delusions. He attempted to abscond, became aggressive with the staff, and was consequently started on Olanzapine dispersible tablet 5 mg once at bedtime.

Later, a more cooperative reassessment revealed that the patient initially experienced a delusional mood with a misinterpretation of phone glitches as peculiar signs. Delusions of reference started to develop and later progressed to persecutory delusions about being followed by the police. Delusions of perception were also described, where normal phenomena such as the sound of knocking were seen as a sign that God was warning him.

Pre-morbidly, the patient was described by his collaterals as a well-adjusted and hardworking individual with a desire to become more health conscious. He reported previously smoking 20 to 40 cigarettes per day for the past six years. Cannabis was consumed socially, with the last use being five months prior to his admission. He denied the use of any other illicit substances and reported alcohol consumption as occurring only on social occasions.

Fifteen days prior to his presentation, the patient had been enrolled in a smoking cessation clinic after fulfilling the criteria for a tobacco use disorder of moderate severity. He was subsequently started on a nicotine patch and received varenicline 0.5 mg twice daily. The dose was increased to 1 mg twice daily after three days as a pharmacological adjunct in his endeavor to quit smoking.

Shortly after initiating the medication, the patient expressed that he had noticed himself becoming very short-tempered, especially towards minor daily inconveniences. He recounted a daily reduction of approximately three hours in his sleep and attributed these changes to an overall increase in workload and stress.

Upon admission to the adult male psychiatry ward, the assessment documented the patient to be alert and oriented, with a calm and cooperative attitude. He requested a prompt discharge after denying having any psychotic symptoms. When asked to share his full recollection of the incident, some propositions were put forward in an attempt to rationalize the reported reckless behavior.

The Olanzapine was suspended with the aim of observing the patient while off psychotropics. Up till then, two doses of the 5 mg Olanzapine had been consumed over the previous two consecutive days while residing in the emergency department. 

After four days, the patient was deemed mentally fit for discharge, and a differential diagnosis of "substance-medication-induced psychotic episode" versus a "brief psychotic episode without a marked stressor," was determined.

Exactly one-week post-discharge, the patient was brought back to the emergency department by his father due to noted suspicious behavior at home and physical aggression. Varenicline was discontinued after the first admission and never re-initiated as per the patient.

The patient was restarted on Olanzapine 5 mg and re-admitted to the psychiatry ward for further assessment. Although the patient continued to demand discharge, he displayed clear paranoid delusions and delusions of reference during his stay in the unit. Olanzapine was accordingly increased to 10 mg daily. The patient denied the use of illicit substances or alcohol, which was reflected in his laboratory investigations. After four days of admission, the patient’s symptoms subsided.

## Discussion

In the case we presented, our patient had no pre-existing psychiatric disorder or family history of psychosis or suicide. He presented after two weeks of initiation of varenicline for the acute onset of persecutory delusions and disorganized behavior. Before admission to inpatient psychiatry, substance-induced psychotic disorders and delirium were ruled out. Varenicline treatment was suspended, after which the patient’s symptoms resolved. The patient scored three on the Naranjo Adverse Drug Reaction Probability Scale done by two physicians involved in the patient’s treatment independently [10]. Thus, the probability was considered possible.

Arteaga et al. describe the case of a 33-year-old man with no significant history who presented with delusions and disorganized speech in the context of the withdrawal of varenicline rather than the initiation of treatment. The patient improved after 19 days of treatment with high doses of risperidone [9]. Another case described by Tuman was similar in terms of age and history; however, the dose of varenicline used, 2 mg twice daily, was double the maximum recommended dose [11]. Other cases are illustrated in Table 1 for reference.

**Table 1:** Case reports of psychosis in the context of treatment with varenicline

Authors	Age	Significant history	Current episode	Onset of symptoms with respect to varenicline treatment	Varenicline treatment	Psychiatric treatment	Duration of symptom
Arteaga et al. [9]	33	No personal or family hx	Delusions and disorganized speech	4 days after cessation	0.5 mg daily for 3 days, 1 mg daily for 1 day	10 mg risperidone	19 days
Tuman [10]	23	No personal or family hx	Persecutory delusions	20 days after initiation	2 mg twice daily	4 mg risperidone	>2 weeks
Forcen et al. [11]	48	No personal hx	Persecutory delusions	second week of initiation	0.5 mg daily then 0.5 mg twice daily for 1 week, 1 mg twice daily	lorazepam 1 mg every 8 hours as needed + zolpidem 10 mg once at night as needed	6 weeks
Gupta et al. [12]	42	Schizophrenia	Breakthrough of psychosis	5-6 days after initiation	0.5 mg daily for 3 days, 1 mg daily for 4 days, 2 mg daily	Same treatment	1 week
Annagur et al. [17]	47	Bipolar disorder	Depressive episode with psychotic features	10 days after initiation	1 mg daily for 1 week, 2 mg daily	Quetiapine-XR 600 mg/day	3 weeks
Knibbs et al. [18]	42	Bipolar disorder	Mania with psychotic features	10 days after initiation	Reaching 1 mg twice daily by day 8	Same treatment	2 days
DiPaula et al. [19]	45	Bipolar disorder	Relapse of delusions and thought broadcasting	2 days after initiation	0.5 mg daily then 0.5 mg twice daily for 1 week, 1 mg twice daily	Increased Olanzapine to 20 mg and Lamotrigine to 125 mg + trifluoperazine 10 mg	>1 month

It is challenging to examine, in our case and in the above-mentioned cases, the stability of the diagnosis, i.e., varenicline-induced psychotic disorder, as patients were not followed up prospectively to determine the existence of an underlying primary psychotic disorder. Gupta et al., for example, reported a patient with preexisting schizophrenia, stable on 25 mg risperidone intramuscular (IM) injection fortnightly that had a breakthrough psychosis five days after the initiation of varenicline. The patient returned to her baseline after stopping varenicline without changing her initial medication regimen [12].

Our patient was readmitted for the re-emergence of his psychotic symptoms briefly after discharge from the mental health hospital. Whether the symptoms remitted completely and reappeared after discharge or were only partially resolved with added efforts to be concealed by the patient, it remains unclear. To the best of our knowledge, there have not been any reported cases of fluctuating psychotic symptoms in the context of varenicline treatment. In our opinion, it was a continuous first episode of psychosis rather than a relapse. During further interviews, it became apparent that our patient was, in fact, guarded and able to mask his symptoms successfully.

In view of the acuteness of his symptoms and their fast resolution after admission, we have also considered the possibility of illicit drug use that was not detected by our routine urine illicit drug screen, i.e., synthetic cannabinoids or salvia divinorum. This could provide an alternate explanation for the remitting-relapsing nature of his presentation. The patient, nonetheless, denies the use of any illicit drugs during the past four months.

Last but not least, an underlying primary psychotic illness remains in the differential diagnosis, i.e., a diagnosis of brief psychotic disorder considering the brevity of the patient’s symptoms. Also, a diagnosis of schizophrenia remains a possibility, previously identified as paranoid type, that can present acutely around the patient’s age, with predominantly positive symptoms and less affective, cognitive, and functional decline. The patient was referred to outpatient psychiatry for further assessment and follow-up.

Ever since varenicline was approved, heterogenous results linking its use with a variety of neuropsychiatric illnesses have emerged. Many cases of psychotic episodes in the context of initiation or withdrawal of varenicline therapy have been reported, both in patients with and without pre-existing psychiatric disorders [13]. In a systematic review of 25 case reports, 68% of patients had a psychiatric history. In 84% of cases, the neuropsychiatric symptoms resolved after the discontinuation of varenicline, and using the Naranjo causality scale, a probable causality was exhibited in 76% of the cases with a definite causality established in 12% of the cases [14]. In a retrospective cohort study conducted in the United States comparing the use of the nicotine patch and varenicline, there was only an associated increase in outpatient clinic visits by patients with pre-existing schizophrenia on varenicline [15]. On the contrary, other case series showed the tolerability and efficacy of varenicline in 15 patients with pre-existing psychotic disorders, specifically schizophrenia [16]. On another note, in a population-based cohort study in Sweden, patients with pre-existing psychiatric diagnoses had an increased risk specific to anxiety (hazard ratio [HR] 1.23, 95% confidence interval [CI] 1.01 to 1.51) and mood conditions (HR 1.31, 95% CI 1.06 to 1.63) after the use of varenicline [15]. In another cohort study done in the UK, it was hard to distinguish whether anxiety development was secondary to the use of varenicline or smoking cessation per se [16]. A definite causal relationship between varenicline and the emergence or aggravation of psychotic symptoms can thus only be speculated.

## Conclusions

The current evidence that links varenicline to psychosis remains controversial, especially in patients with pre-existing psychiatric disorders, as most of the randomized controlled studies excluded patients with pre-existing psychiatric conditions. In addition, most of the studies examined instead mood symptoms, anxiety, and suicide. Speculatively, there could be a link between varenicline, which supposedly increases dopamine levels in the prefrontal cortex through the mesolimbic pathways, and psychotic symptoms. Whether the symptoms are dose-related, occur during initiation of treatment or cessation, and whether people with pre-existing psychiatric illnesses are more sensitive to this rise in dopamine, remains unclear. It is nonetheless beneficial to be cognizant, in a clinical setting, of the possibility of the emergence of these symptoms with varenicline therapy.
